# A Rural Tele-Video Model: Results From Educating Practitioners About Opioids and Alternative Pain Management

**DOI:** 10.1093/geroni/igaa057.620

**Published:** 2020-12-16

**Authors:** Leah Tobey, Robin McAtee, Corey Hayes

**Affiliations:** 1 University of Arkansas for Medical Sciences, Little Rock, Arkansas, United States; 2 UAMS, Little Rock, Arkansas, United States

## Abstract

Providers in Arkansas wrote 105.4 opioid prescriptions for every 100 persons in 2017-nearly twofold greater than the average U.S. rate of 58.7 opioid prescriptions (CDC, 2017). This makes AR the second highest opioid prescribing state. AR-IMPACT (Arkansas Improving Multi-Disciplinary Pain Care and Treatment) is a tele-video case conference education model which was designed to improve AR providers’ knowledge and behavior in pain management and improve awareness of opioid-sparing alternatives. Conference panelists include a geriatrician, psychiatrist, physical therapist, psychologist, pharmacist, a chronic pain specialist and various guest speakers such as the Arkansas Drug Director. During weekly tele-video conferences, relevant actionable cases were presented regarding treating diverse patients who have chronic pain. AR-IMPACT provided hundreds of practitioners with up-to-date data and education regarding opioid use and misuse, methods and resources regarding titrating and eliminating opioids, and viable alternative pain-management solutions such as motivational interviewing and dry-needling. This poster will present the background of the opioid crisis in rural Arkansas followed by the specifics of the development and application of the AR-IMPACT tele-video model. Specific older adult cases studies, quantitative and qualitative outcomes, feedback from presenters and participants, and lessons learned will be reviewed. Specifically, lessons learned center around the practitioners’ culture of pain management and how that culture must evolve and change. New methods of pain management must be taught to practitioners and patients and adopted as the new norm. This will demonstrate that programs like AR-IMPACT are crucial in helping make that cultural change happen.

